# Increased expression of the tight junction protein TJP1/ZO-1 is associated with upregulation of TAZ-TEAD activity and an adult tissue stem cell signature in carfilzomib-resistant multiple myeloma cells and high-risk multiple myeloma patients

**DOI:** 10.18632/oncoscience.356

**Published:** 2017-08-01

**Authors:** Irene Riz, Robert G. Hawley

**Affiliations:** ^1^ Department of Anatomy and Regenerative Biology, George Washington University, Washington, DC, USA

**Keywords:** multiple myeloma, proteasome inhibitors, carfilzomib, cancer stem cell-related features, TJP1/ZO-1, WWTR1/TAZ-TEAD1, translation inhibitors, homoharringtonine, Nrf2

## Abstract

Tight junction protein 1 (TJP1) has recently been proposed as a biomarker to identify multiple myeloma (MM) patients most likely to respond to bortezomib- and carfilzomib-based proteasome inhibitor regimens. Herein we report increased expression of *TJP1* during the adaptive response mediating carfilzomib resistance in the LP-1/Cfz MM cell line. Moreover, increased *TJP1* expression delineated a subset of relapsed/refractory MM patients on bortezomib-based therapy sharing an LP-1/Cfz-like phenotype characterized by activation of interacting transcriptional effectors of the Hippo signaling cascade (TAZ and TEAD1) and an adult tissue stem cell signature. siRNA-mediated knockdown of TJP1 or TAZ/TEAD1 partially sensitized LP-1/Cfz cells to carfilzomib. Connectivity Map analysis identified translation inhibitors as candidate therapeutic agents targeting this molecular phenotype. We confirmed this prediction by showing that homoharringtonine (omacetaxine mepesuccinate) — the first translation inhibitor to be approved by the U.S. Food and Drug Administration — displayed potent cytotoxic activity on LP-1/Cfz cells. Homoharringtonine treatment reduced the levels of TAZ and TEAD1 as well as the MM-protective proteins Nrf2 and MCL1. Thus, our data suggest the importance of further studies evaluating translation inhibitors in relapsed/refractory MM. On the other hand, use of *TJP1* as a MM biomarker for proteasome inhibitor sensitivity requires careful consideration.

## INTRODUCTION

Over the past decade, the prognosis of multiple myeloma (MM) patients has improved with the introduction of new drugs such as the first- and second- generation proteasome inhibitors bortezomib and carfilzomib [1]. Unfortunately, however, MM patients routinely develop resistance to proteasome inhibitor-based therapies and eventually succumb to their disease [2-5]. Toward identification of novel agents that will overcome resistance to this class of anti-MM drugs, we have developed isogenic models of carfilzomib resistance using a panel of patient-derived MM cell lines [6-9]. Our studies have revealed upregulated expression of the ABCB1/P-glycoprotein multidrug resistance efflux transporter, elevated prosurvival autophagy and antioxidant defense, altered intermediary metabolism and reprogramming of the translation machinery as key processes mediating acquired resistance to carfilzomib [6-9].

Recently, Orlowski and colleagues reported that relative resistance to bortezomib and carfilzomib in some MM cell lines was associated with decreased expression of the tight junction protein 1 gene *TJP1* (also known as zonula occludens 1, ZO-1) and they proposed that high *TJP1* expression might be used as a biomarker of proteasome inhibitor sensitivity in the clinic [10]. In line with this, we observed that TJP1 transcript levels were decreased in two of our carfilzomib-resistant MM cell lines compared to their parental counterparts (KMS-11/Cfz and KMS-34/Cfz versus KMS-11 and KMS-34 cells, respectively; GEO: GSE69078). In contrast, we noted that carfilzomib-adapted LP-1/Cfz cells — also cross-resistant to bortezomib — expressed higher TJP1 transcript levels than parental LP-1 cells (GEO: GSE78069) [8].

Here we confirm that TJP1 protein levels are increased in LP-1/Cfz cells. Moreover, increased *TJP1* expression delineated a subset of relapsed/refractory MM patients on bortezomib-based therapy [11] sharing an LP-1/Cfz-like phenotype characterized by an adult tissue stem cell signature [12] and activation of interacting transcriptional effectors of the Hippo signaling cascade: TAZ (transcriptional co-activator with PDZ-binding motif encoded by the WWTR1 gene) and TEAD1 (TEA domain transcription factor 1) [13-16].

TAZ shares ~50% identity with YAP1 (Yes associated protein 1), another downstream effector of the Hippo pathway that intriguingly had previously been found to be homozygously deleted or generally downregulated in MM [17]. There are several structural differences between TAZ and YAP1 that are likely related to their overlapping yet distinct functional properties [13, 18]. Furthermore, it is becoming increasingly appreciated that TAZ activity is regulated by multiple inputs in addition to the Hippo kinase cascade, including cell morphology and mechanical cues from the extracellular microenvironment [19, 20].

siRNA-mediated knockdown of TJP1 or TAZ/TEAD1 partially sensitized LP-1/Cfz cells to carfilzomib. Our *in vitro* findings were supported by an independent clinical data set [21] where MM patients with the LP-1/Cfz-like molecular phenotype — i.e, high *TJP1, WWTR1/TAZ* and *TEAD1* expression — was associated with inferior overall survival outcomes.

To identify novel agents that would potentially overcome resistance to this class of anti-MM drugs, we performed Connectivity Map (CMap) analysis [22] and uncovered translation inhibitors whose gene expression perturbations were significantly anticorrelated with the expression signatures shared by LP-1/Cfz cells and the relapsed/refractory MM cases with increased *TJP1* expression. We confirmed the CMap prediction by showing that homoharringtonine (omacetaxine mepesuccinate) — the first translation inhibitor to be approved by the U.S. Food and Drug Administration — displayed potent cytotoxic activity on LP-1/Cfz cells. Cytotoxicity was associated with decreased TAZ and TEAD1 protein levels as well as two proteins, Nrf2 and MCL1, previously identified by us and others as contributing to MM drug resistance [8, 9, 23-25].

## RESULTS AND DISCUSSION

### TJP1 is associated with drug resistance in LP-1/Cfz and RPMI-8226/Dox40 MM cells

In prior work, we found that the transcription factor NF-E2 p45-related factor 2 (Nrf2; gene symbol *NFE2L2*) contributes to the carfilzomib-resistant phenotype in LP-1/Cfz cells characterized by an epithelial-to-mesenchymal transition (EMT)-like expression signature [8]. EMT is a developmental program that is often activated during tumor metastasis resulting in the gain of stem cell properties by the malignant cells [26]. During developmental EMT, epithelial intercellular junctions are disrupted and *TJP1* is coordinately downregulated with *CDH1* (E-cadherin) [27]. Cell surface expression of E-cadherin was decreased on LP-1/Cfz cells compared to parental LP-1 cells [8], but TJP1 protein levels were predicted to be ~2-fold increased (Table S1: Expression changes, TJP1 202011_at probe set). Of potential relevance in this regard, upregulation of TJP1 has been associated with invasion and metastasis in certain tumor systems [28-30].

**Figure 1:**
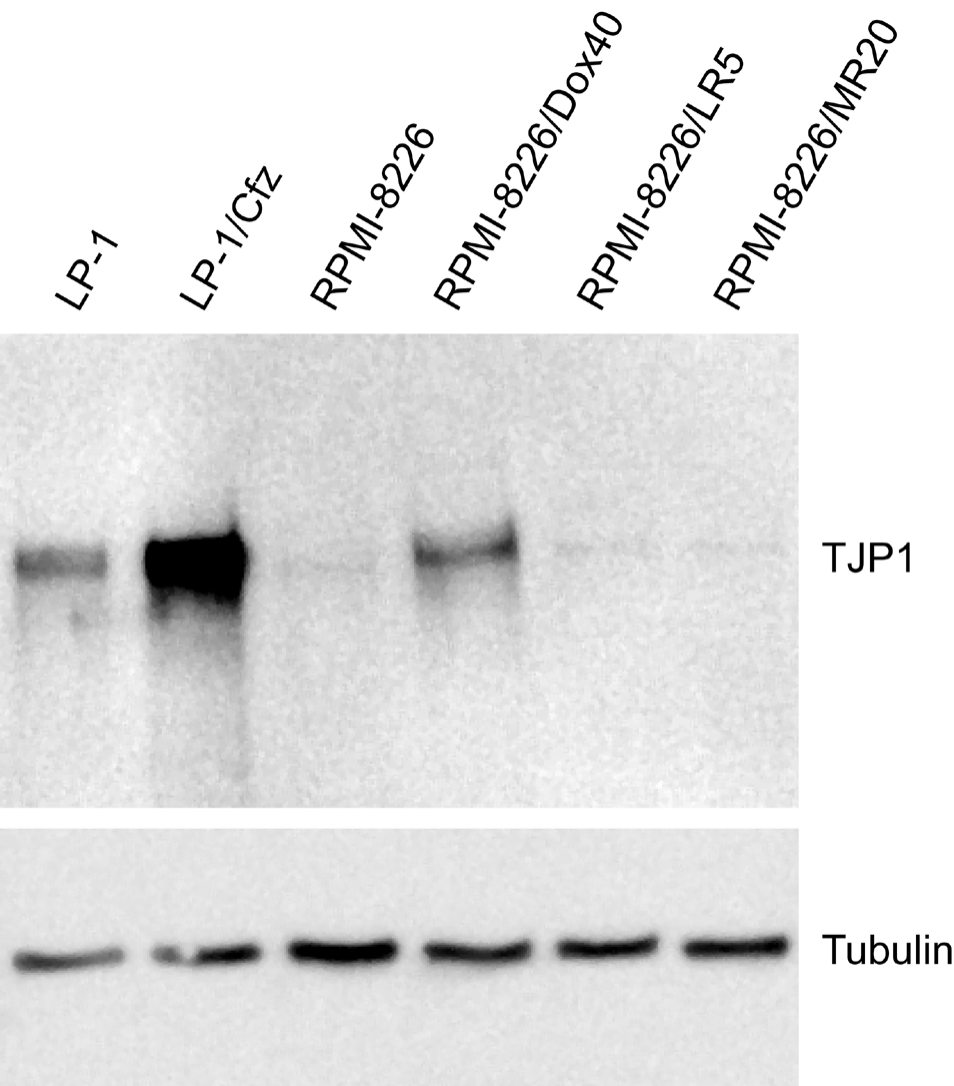
TJP1 protein levels are increased in carfilzomib-resistant LP-1/Cfz cells Western blot analysis showing significantly higher TJP1 levels in LP-1/Cfz compared to parental LP-1 cells. Note that TJP1 levels are also increased in doxorubicin-resistant RPMI-8226/Dox40 compared to parental RPMI-8226 cells.

Western blot analysis showed significantly higher TJP1 levels in LP-1/Cfz compared to parental LP-1 cells (Figure 1). For comparison, we also examined TJP1 levels in RPMI-8226 MM cells analyzed by Orlowski and colleagues [10] together with three drug-resistant RPMI-8226 derivatives: RPMI-8226/Dox40 cells, selected for resistance to doxorubicin [31]; RPMI-8226/LR5 cells, selected for resistance to melphalan [32]; and RPMI-8226/MR20 cells, selected for resistance to mitoxantrone [33]. TJP1 levels were increased in RPMI-8226/Dox40 cells; however, no significant changes were observed in the other derivatives (Figure 1). This was noteworthy because we and others have shown that RPMI-8226/Dox40 cells are cross-resistant to both carfilzomib and bortezomib due in part to upregulation of ABCB1/P-glycoprotein [6, 34]. These results indicated that overexpression of TJP1 in MM cells is not universally associated with increased sensitivity to proteasome inhibitors. Consequently, we were interested in determining whether there were instances where MM patients who developed resistance to proteasome inhibitor-based therapies also exhibited increased *TJP1* expression and whether they might share similar properties with LP-1/Cfz or RPMI-8226/Dox40 cells.

### Increased TJP1 expression during disease progression identifies a subset of relapsed/refractory MM patients on bortezomib-based therapy exhibiting an LP-1/Cfz-like phenotype

We first examined *TJP1* levels in the GEO: GSE31161 data set that contains expression profiling data at diagnosis and relapse for MM patients treated with the University of Arkansas for Medical Sciences (UAMS) Total Therapy (TT3) protocol which incorporates bortezomib up-front into the Total Therapy 2 (TT2) tandem transplant regimen [11]. Out of 30 evaluable patients, 13 (43.3%) had significantly higher *TJP1* expression at relapse (TJP1-up patients: ~6.5-fold up; log FC for the TJP1 202011_at probe set, 2.70; P = 7.63× 10-4) and 17 (56.7%) had significantly lower *TJP1* expression at relapse (TJP1-down patients: ~3.7-fold down; log_2_ FC for the TJP1 202011_at probe set, -1.88; P = 3.98 × 10-3) (Table S1: Expression changes). Neither group had increased ABCB1 expression at relapse (Table S1: Expression changes, ABCB1 209993_at probe set).

**Figure 2:**
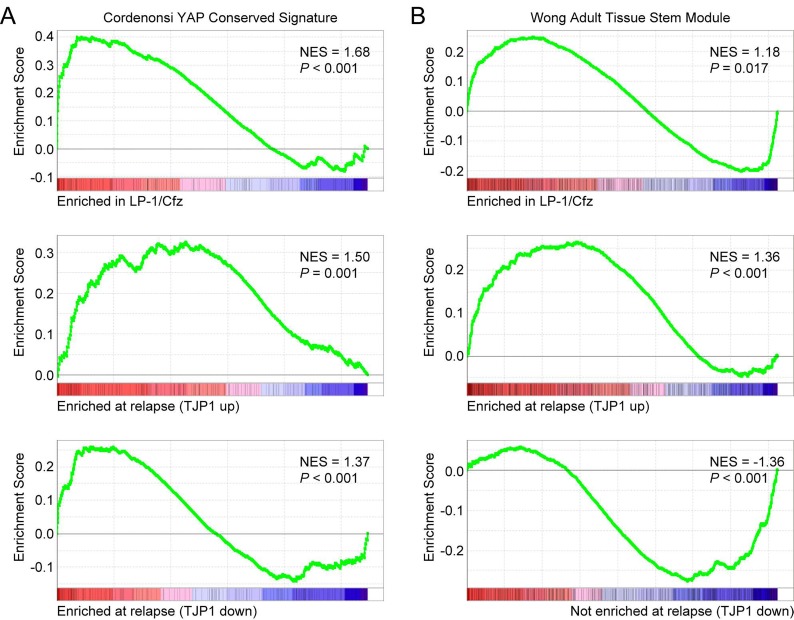
Increased expression of TJP1 is associated with upregulation of TAZ-TEAD activity and adult stem cell genes in LP-1/Cfz cells and TT3 protocol patients with relapsed/refractory disease (GEO: GSE31161) A. GSEA enrichment plots of the Cordenonsi YAP Conserved Signature for triplicate samples of LP-1/Cfz versus parental LP-1 cells (top), for TT3 patients with increased TJP1 levels at relapse (middle), and for TT3 patients with decreased TJP1 levels at relapse (bottom). B. GSEA enrichment plots of the Wong Adult Tissue Stem Module for triplicate samples of LP-1/Cfz versus parental LP-1 cells (top), for TT3 patients with increased TJP1 levels at relapse (middle), and for TT3 patients with decreased TJP1 levels at relapse (bottom). NES, normalized enrichment score.

Next we applied Gene Set Enrichment Analysis (GSEA) to compare the two TT3 patient subsets with LP-1/Cfz cells [35]. Similar to LP-1/Cfz cells, significant enrichment of an EMT-like signature (Hallmark Epithelial Mesenchymal Transition) was observed for TJP1-up patients (normalized enrichment score [NES] = 1.78, P< 0.001) (Table S1: Hallmark collection). Also, as with LP-1/Cfz cells, significant enrichment of an Nrf2 target gene set (NFE2L2.V2) was found for TJP1-up patients (NES = 1.43, P < 0.001) (Table S1: C6 Oncogenic signatures). Interestingly, LP-1/Cfz cells and both TT3 patient subsets showed enrichment of the Cordenonsi YAP Conserved Signature associated with cancer stem cell-related properties (Figure 2A; Table S1: C6 Oncogenic signatures). Notably, among the 3,400 gene sets in the C2.CGP Chemical and genetic perturbations collection, the Wong Adult Tissue Stem Module, containing genes coordinately upregulated in a compendium of adult tissue stem cells [12], was enriched in both LP-1/Cfz cells and TJP1-up patients (Figure 2B). Conversely, TJP1-down patients had negative enrichment of this gene set (Figure 2B) and instead had enrichment of an embryonic stem cell-like signature (Wong Embryonic Stem Cell Core; NES = 3.42; P < 0.001) [12]. Collectively, the results indicated that the 30 MM cases that progressed on bortezomib-based TT3 therapy had acquired transcriptional programs shared with stem cells. Increased *TJP1* expression delineated a TT3 subset most closely resembling LP-1/Cfz cells: i.e., exhibiting EMT-like features, Nrf2 activation and enrichment of an adult tissue stem cell signature.

### Elevated TAZ and TEAD1 protein levels provide an explanation for the Cordenonsi YAP Conserved Signature in LP-1/Cfz cells

**Figure 3:**
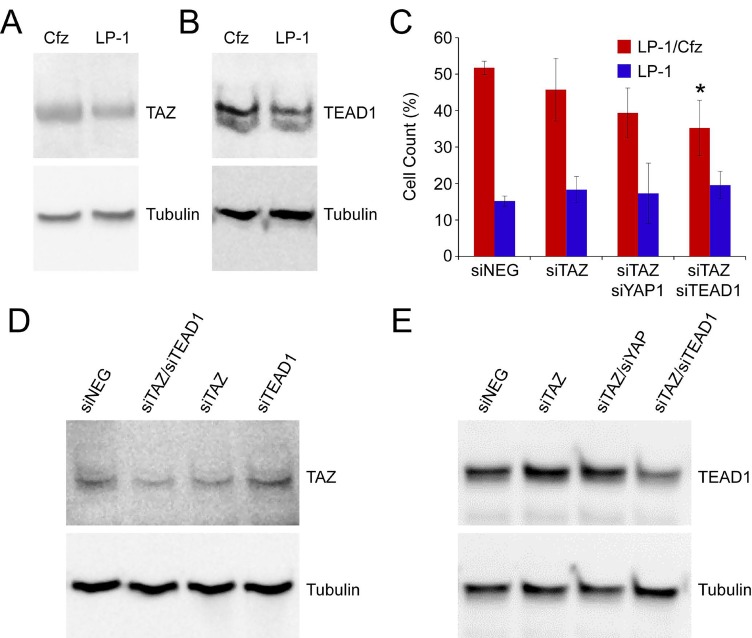
TAZ-TEAD1 activation in LP-1/Cfz cells confers resistance to carfilzomib A. TAZ levels are increased in LP-1/Cfz (Cfz) versus parental LP-1 cells (1.6 ± 0.3-fold, n = 10, P < 0.01). Cells were pretreated with MG-132 (15 μM) for 18 hours to prevent proteasomal degradation. B. TEAD1 levels are increased in LP-1/Cfz (Cfz) versus parental LP-1 cells (2.2 ± 0.3-fold, n = 10, P < 0.01). Cells were pretreated with MG-132 (15 μM) for 18 hours to prevent proteasomal degradation. C. siRNA-mediated knockdown of TAZ slightly sensitized LP-1/Cfz cells to carfilzomib. Consistent with low YAP1 levels in LP-1/Cfz cells, siRNA-mediated knockdown of YAP1 in concert with TAZ did not meaningfully enhance sensitization of the cells to the drug. LP-1/Cfz cells were significantly sensitized to carfilzomib by combined siRNA-mediated knockdown of TAZ and TEAD1. No effects were observed on parental LP-1 cells under any of the conditions. Cells were treated with carfilzomib (12.5 nM) for 48 hours after transient transfection and cell viability was determined by alamarBlue assay. *, P = 0.04 vs negative siRNA control (siNeg, n = 3). D, E. siRNA-mediated knockdown of TAZ and TEAD1 in LP-1/Cfz cells was accompanied by decreased TAZ (40 ± 15% decrease) (D) and TEAD1 (57 ± 6% decrease) (E) levels.

The Cordenonsi YAP Conserved Signature, representing a list of evolutionary conserved target genes of the YAP1 transcriptional effector of the Hippo signaling pathway, was the top-ranked gene set for LP-1/Cfz cells (Table S1: C6 Oncogenic signatures). We had not focused on it earlier because, as previously reported for most MM and other hematopoietic cancer cells [17], YAP1 mRNA levels were low in LP-1 cells and were not increased in LP-1/Cfz cells (~1.2-fold down; log FC for the YAP1 213342_at probe set, -0.24; P = 0.14) (Table S1: Expression changes). Upon further investigation, we learned that the Cordenonsi YAP Conserved Signature was enriched in breast cancer cells undergoing EMT that resulted in the activation of TAZ, a paralog of YAP1, which conferred stem cell-like properties on the tumor cells [36]. Slightly increased expression of the TAZ-encoding gene *WWTR1* was observed in LP-1/Cfz cells (~1.3-fold up; log FC for the WWTR1 202133_at probe set, 0.41; P = 0.05) (Table S1: Expression changes). In addition, *TEAD1*, a key DNA-binding transcription factor partner of TAZ [14, 15], was highly expressed in both LP-1 and LP-1/Cfz cells (TEAD1 224955_at probe set; GEO: GSE78069). As anticipated [17], YAP1 protein levels were low in LP-1 cells (below detection by western blotting). Although WWTR1/TAZ mRNA levels were comparable to YAP1 mRNA levels (GEO: GSE78069), TAZ protein could be detected in LP-1 cells and elevated levels were observed in LP-1/Cfz cells (Figure 3A). Strikingly, TEAD1 protein levels were also markedly increased in LP-1/Cfz cells (Figure 3B). These findings were reminiscent of the translational upregulation we previously documented for Nrf2 in LP-1/Cfz cells [8, 9]. Importantly, combined knockdown of TAZ and TEAD1 with previously validated siRNAs [37, 38] partially sensitized LP-1/Cfz cells to carfilzomib (P = 0.04; Figure 3C-E).

### Increased *TJP1* expression separates out MM patients with poor prognosis into a higher-risk LP-1/Cfz-like subset

**Figure 4:**
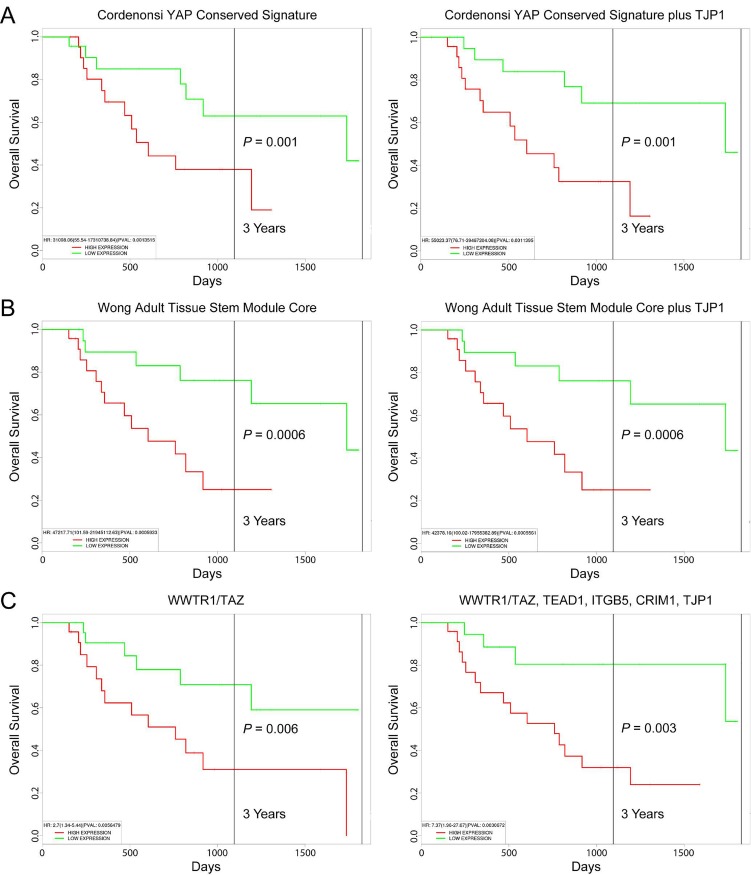
Prognostic value of TJP1 expression in MM patient survival outcomes KaplanMeier survival plots of 47 MM patients in the high-risk PR subgroup (GEO: GSE2658) created using PROGgeneV2: A. Cordenonsi YAP Conserved Signature minus (left) and plus (right) TJP1 coexpression. B. Leading edge subset (top 50 genes) of the LP-1/Cfz Wong Adult Tissue Stem Module minus (left) and plus (right) TJP1 coexpression. C. WWTR1/TAZ expression (left) plus coexpression of TEAD1, ITGB5, CRIM1 and TJP1 (right). Median gene expression values were used as bifurcation points.

Acquisition of EMT-like features was previously shown to promote the extramedullary dissemination of MM cells [39]. Gene profiling of extramedullary MM has revealed differential expression of adhesion molecules and factors that stimulate angiogenesis [40]. In this regard, significant enrichment of an angiogenesis signature (Hallmark Angiogenesis) was observed for both LP-1/Cfz cells and for TJP1-up patients (NES = 1.68, P < 0.001 and NES = 1.92, P < 0.001, respectively) (Table S1: Hallmark collection). Moreover, we noted that *WWTR1/TAZ* was included among the 156 upregulated genes in extramedullary MM [40]. A separate gene expression profiling study conducted by the UAMS group showed that extramedullary MM was more prevalent in patients with high-risk disease that includes those in the proliferation-related “PR” subgroup [41]. The PR subgroup was previously defined in an independent cohort of newly diagnosed patients treated on UAMS TT2 and TT3 protocols (GEO: GSE2658) [21]. We used the PROGgeneV2 prognostic biomarker identification tool to investigate the impact of *TJP1* expression on the overall survival outcomes of MM patients in the GEO: GSE2658 data set [42]. Using median gene expression values as bifurcation points, Cox proportional hazards regression analysis showed that MM patients in the PR subgroup with the Cordenonsi YAP Conserved Signature (57 genes) had significantly inferior overall survival, with worse prognosis when high level *TJP1* coexpression was also considered (Figure 4A). High expression of the top 50 genes in the leading edge subset of the LP-1/Cfz Wong Adult Tissue Stem Module (721 genes total) was also associated with adverse outcomes. Within these parameters, *TJP1* expression levels did not provide any additional prognostic information (Figure 4B).

Only 2 out of the top 50 genes in the leading edge subset of the LP-1/Cfz Wong Adult Tissue Stem Module are shared with the Cordenonsi YAP Conserved Signature: *ITGB5* and *CRIM1*, which were also among the 156 upregulated genes in extramedullary MM [40]. *ITGB5*, encoding β5-integrin, was previously demonstrated to play a critical role in EMT associated with the tumorigenic potential of breast cancer cells [43]. *CRIM1* encodes cysteine rich transmembrane bone morphogenetic protein regulator 1, which has been shown to regulate integrin signaling and angiogenesis during development [44, 45]. Increased expression of both genes has been associated with the acquisition of chemoresistance [46, 47]. Notably, *ITGB5* and *CRIM1* are coexpressed with *WWTR1*/*TAZ* (top 20 gene neighbors) across 947 cell lines in the Cancer Cell Line Encyclopedia (CCLE; Figure S1) [48]. Furthermore, *TJP1* is among the top 20 gene neighbors of both *TEAD1* and *ITGB5* in the CCLE (Figure S1). Kaplan-Meier plots indicated significant segregation in survival outcomes for patients in the PR subgroup with high versus low *WWTR1/TAZ* expression (hazard ratio, HR = 2.7; 95% confidence interval/CI, 1.34-5.44; P = 0.006). Increasing risk of mortality was observed when high level coexpression of *TEAD1, ITGB5* and *CRIM1* (HR = 3.98; 95% CI, 1.63-9.68; P = 0.002) and *TEAD1, ITGB5, CRIM1* plus *TJP1* were also taken into consideration (HR = 7.37; 95% CI, 1.96-27.67; P = 0.003) (Figure 4C). It should be mentioned that high level expression of *TEAD1* had previously been associated with high-risk disease and the PR subgroup in MM [49]. However, the biological relevance of this observation has not been heretofore appreciated.

### Increased *TJP1* expression contributes to LP-1/Cfz-like high-risk MM phenotype and carfilzomib resistance

**Figure 5:**
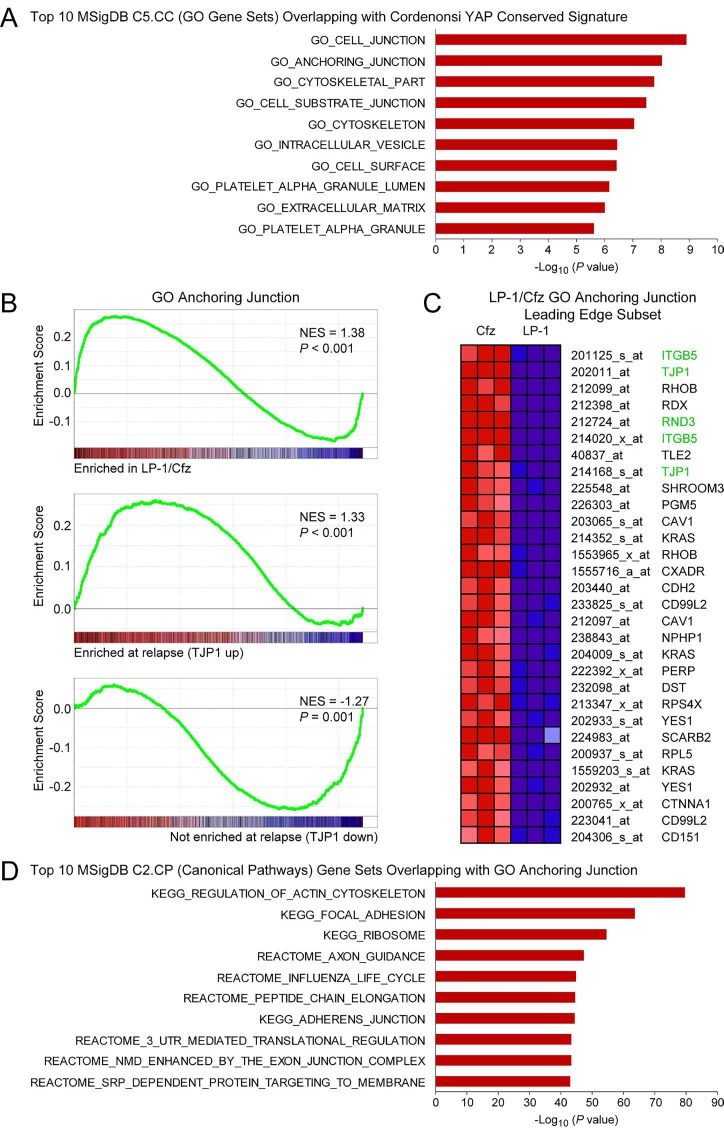
Gene Ontology terms and canonical pathways involving cell contacts are enriched in LP-1/Cfz cells and TT3 protocol patients (GEO: GSE31161) with increased TJP1 levels at relapse A. Bar graphs showing the top 10 gene sets out of 580 total gene sets in the MSigDB C5.CC Gene Ontology Cellular Component collection that overlap with the Cordenonsi YAP Conserved Signature. P values were calculated using the hypergeometric distribution. See also Table S1: GO gene sets. B. GSEA enrichment plots of the GO Anchoring Junction gene set for triplicate samples of LP-1/Cfz versus parental LP-1 cells (top), for TT3 patients with increased TJP1 levels at relapse (middle), and for TT3 patients with decreased TJP1 levels at relapse (bottom). NES, normalized enrichment score. C. Heat map of the leading edge subset of genes in the GO Anchoring Junction gene set upregulated in LP-1/Cfz (Cfz) versus parental LP-1 cells (triplicate samples). Probe sets for TJP1, ITGB5 and RND3 are highlighted. See text for details. D. Bar graphs showing the top 10 gene sets out of 1329 total gene sets in the MSigDB C2.CP Canonical pathways collection that overlap with the GO Anchoring Junction gene set. Note that 5 out of the 10 gene sets involve translational control mechanisms. P values were calculated using the hypergeometric distribution. See also Table S1: C2.CP Canonical pathways.

Although *TJP1* was not identified as a YAP/TAZ target gene in the Cordenonsi YAP Conserved Signature, it was a member of the top two overlapping gene sets in the MSigDB C5.CC Gene Ontology Cellular Component collection (580 total gene sets): GO Cell Junction (13 out of 57 genes; P = 1.26 × 10-9) and GO Anchoring Junction (9 out of 57 genes; P = 9.30 × 10-9) (Figure 5A). Both gene sets were selectively enriched in LP-1/Cfz cells and TJP1-up patients (Figure 5B; Table S1: C5 GO gene sets). Conversely, TJP1-down patients had negative enrichment of these gene sets (Figure 5B; and data not shown). Moreover, ITGB5 was included with *TJP1* in the LP-1/Cfz leading edge subset of the GO Anchoring Junction gene set (Figure 5C). Indeed, enrichment of canonical pathways and GO-term gene sets involving cell contacts — previously noted to be overrepresented within the TAZ interactome data set [18] — was observed for LP-1/Cfz cells and TJP1-up patients (Figure 5A,D; Table S1: C2.CP Canonical pathways, C5 GO gene sets), suggesting biological relevance of *TJP1* upregulation [19, 20].

**Figure 6:**
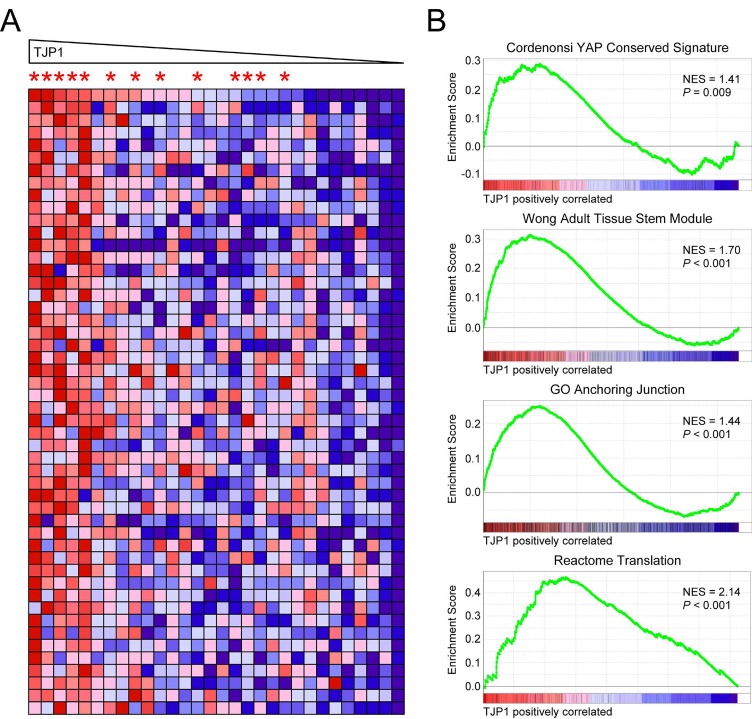
Enriched MSigDB gene sets in MM patients enrolled on the TT3 protocol with high TJP1 levels at relapse A. Heat map of TJP1 neighbors for 30 TT3 relapsed samples in the GEO: GSE31161 data set stratified on the basis of TJP1 202011_at probe set levels. Asterisks indicate TJP1-up patients (see Table S1: GSE31161, TT3 samples). B. Enrichment plots of selected gene sets containing genes whose expression is highly correlated with TJP1 expression. NES, normalized enrichment score.

To further explore the potential functional significance of increased *TJP1* expression with respect to the LP-1/Cfz-like high-risk MM phenotype, we stratified the 30 TT3 relapsed samples in the GEO: GSE31161 data set on the basis of *TJP1* expression (TJP1 202011_at probe set). While the samples did not perfectly segregate into two separate groups, those with the highest TJP1 mRNA levels belonged to the TJP1-up subset (increased *TJP1* expression at relapse) and those with the lowest TJP1 mRNA levels belonged to the TJP1-down subset (decreased *TJP1* expression at relapse) (Figure 6A). When we performed GSEA to identify gene sets that positively correlated with *TJP1* expression, we found that the Cordenonsi YAP Conserved Signature, the Wong Adult Tissue Stem Module and the GO Anchoring Junction gene sets were all significantly enriched (Figure 6B). Significant enrichment of the Cordenonsi YAP Conserved Signature was previously observed after development of chemoresistance in melanoma cells [50]. In that study, in which *TJP1* was one out of 426 significantly upregulated genes common to two cell lines selected for resistance to the BRAF inhibitor PLX4032 (vemurafenib) (see Table EV1 of ref. [50]), increased YAP/TAZ transcriptional activity was accompanied by enhanced YAP/TAZ nuclear localization.

**Figure 7:**
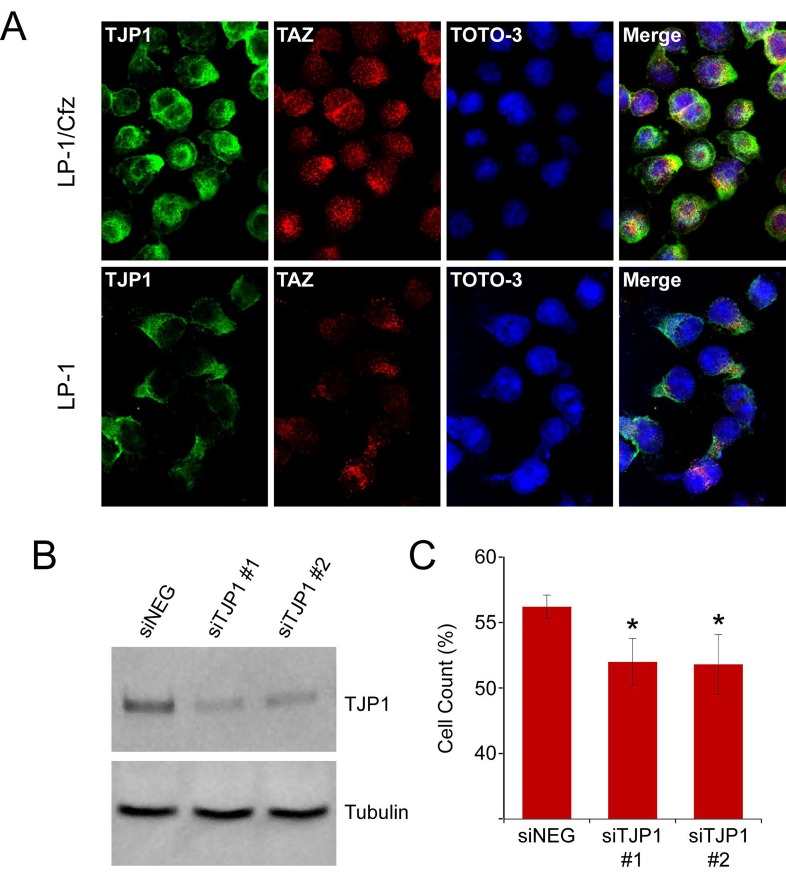
Subcellular localization and knockdown of TJP1 in LP-1/Cfz cells A. LP-1/Cfz and parental LP-1 cells were cultured on poly-L-lysine-coated microscope slides. Cells were fixed and labeled with anti-TJP1 (Alexa Fluor 488, green) or anti-TAZ (Alexa Fluor 568, red) antibodies. Cell nuclei were stained with TOTO-3 (blue). B. siRNA-mediated knockdown of TJP1 mRNA was accompanied by decreased TJP1 levels (64 ± 4% decrease; n = 3). C. Knockdown of TJP1 mRNA using two specific siRNAs (siTJP1 #1, siTJP1 #2) sensitizes LP-1/Cfz cells to carfilzomib. Cells were cultured on fibronectin-coated plates and treated with carfilzomib (6 nM) for 48 hours after transient transfection; cell viability was determined by alamarBlue assay. *, P = 0.03 vs negative siRNA control (siNeg, n = 3).

In line with this, TAZ predominantly localized to the nucleus in a characteristic punctate staining pattern [13] as well as to the perinuclear region of LP-1/Cfz cells (Figure 7A, top panels). Lower levels of TAZ were present in parental LP-1 cells with a greater distribution in the perinuclear region (Figure 7A, bottom panels). By comparison, TJP1 was present in the cytoplasm, plasma membrane and nucleus of LP-1/Cfz cells (Figure 7A, top panels) whereas it was primarily detected in the cytoplasm of parental LP-1 cells (Figure 7A, bottom panels). Some colocalization of TJP1 with TAZ was observed in LP-1/Cfz cells but not within the punctate nuclear structures. These results indicated that, while TJP1 is able to bind to TAZ *in vitro* [51], functional complexes between TJP1 and TAZ are not responsible for TAZ nuclear retention [52, 53].

To directly test whether *TJP1* contributes to carfilzomib resistance, we transfected LP-1/Cfz cells with two previously validated TJP1 siRNAs [54, 55]. We confirmed that both siRNAs resulted in a significant decrease in TJP1 protein levels (64 ± 4% decrease; n =3) (Figure 7B). As illustrated in Figure 7C, knockdown of TJP1 with both siRNAs modestly, but significantly, sensitized LP-1/Cfz cells to carfilzomib (P = 0.03).

### Translation inhibitors as potential treatment for relapsed/refractory MM

**Figure 8:**
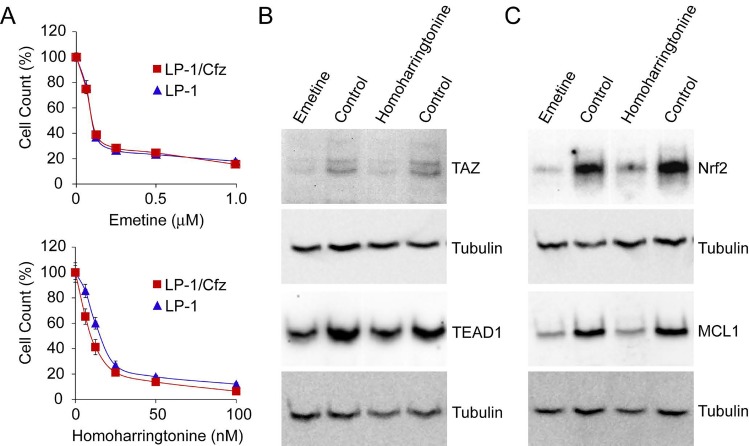
Cytotoxic activity of translation inhibitors against LP-1/Cfz cells A. Cells were treated with the indicated concentrations of emetine (top) or homoharringtonine (bottom) for 48 hours and cell viability was determined by alamarBlue assay. B. Treatment with emetine (0.3 μM) or homoharringtonine (30 nM) for 18 hours decreases TAZ (top) and TEAD1 (bottom) levels in LP-1/Cfz cells. C. Treatment with emetine (0.3 μM) or homoharringtonine (30 nM) for 18 hours decreases Nrf2 (top) and MCL1 (bottom) levels in LP-1/Cfz cells.

Consistent with the post-transcriptional increase in TEAD1 (and, to some degree, TAZ) levels and the translational adaptations that resulted in increased Nrf2 protein levels in LP-1/Cfz cells [8, 9], pathways related to translational control of gene expression were among the overrepresented processes shared with TJP1-up patients (Figures 5D, 6B; Table S1: C2.CP Canonical pathways, C5 GO gene sets). In a complementary approach, when we queried the Connectivity Map database (CMap Build 02), a reference collection of 6,100 gene expression signatures from four human cell lines treated with 1,309 small molecule perturbagens [22], we identified gene expression perturbations by cycloheximide and emetine as the only ones that were significantly negatively correlated with the expression signatures linked to both carfilzomib resistance in LP-1/Cfz cells and the TJP1-up MM cases (P ≤ 0.05) (Table S1: CMap Build 02). These results suggested that translation inhibitors may modulate chemosensitivity of MM cells with these shared features by reversing the expression of the signature genes responsible [56, 57]. We tested emetine and found that it displayed single-agent activity against LP-1/Cfz cells (Figure 8A, top). This result prompted us to examine the effect of the FDA-approved translation inhibitor homoharringtonine (omacetaxine mepesuccinate) [58], which had previously been demonstrated to induce apoptosis but had not been reported to overcome drug resistance in MM cells [59]. Treatment with homoharringtonine displayed potent cytotoxicity against LP-1/Cfz cells over a concentration range that was 10-fold lower than that of emetine (Figure 8A, bottom). During revision of this manuscript, a new version of the CMap database was released that is accessible via CLUE (CMap and LINCS Unified Environment: [clue.io]). The Touchstone dataset of CLUE contains ~50,000 gene expression signatures from nine human cell lines treated with 2,911 FDA-approved and clinical trial drugs. It was especially noteworthy therefore that expression signatures generated by homoharringtonine as well as emetine and cycloheximide in fact, translation inhibitors in general — were among those displaying the most significant negative correlations with the LP-1/Cfz and TJP1-up MM signatures (Table S1: CMap CLUE).

Translation inhibitors induce cell death by reducing the levels of short-lived prosurvival proteins like the BCL2 family member MCL1 [59]. TAZ is an unstable protein with a half-life of ~2 hours in other cell types [60]. Treatment of LP-1/Cfz cells with emetine or homoharringtonine led to reduced amounts of TAZ; the levels of TEAD1, which has a half-life of ~16 hours in fibroblasts [61], were also reduced under the 18-hour treatment conditions employed (Figure 8B). Nrf2 is also a short-lived protein [62], and Nrf2 levels were significantly reduced by both translation inhibitors (Figure 8C). Moreover, recent studies have provided additional support for an important role of MCL1 in MM cell survival [24, 25] (ClinicalTrials.gov: NCT02675452, NCT02992483). Both emetine and homoharringtonine significantly reduced MCL1 levels (Figure 8C).

### Concluding remarks

A recent report by Orlowski and colleagues suggested the use of *TJP1* as a biomarker to identify MM patients most likely to benefit from proteasome inhibitors [10]. The investigators proposed that high TJP1 levels resulted in decreased proteasome activity due to suppression of expression of the *PSMB8* gene encoding the immunoproteasome β5i/LMP7 (chymotrypsin-like) subunit targeted by carfilzomib and bortezomib as well as the *PSMB9* gene encoding the β1i/LMP2 (caspase-like) subunit that is also targeted by bortezomib [10]. This model does not hold for LP-1/Cfz cells nor for the subset of MM patients on bortezomib-based therapy analyzed here. In the case of LP-1/Cfz cells, *PSMB8* and *PSMB9* were downregulated concomitant with increased *TJP1* expression (~1.5-fold; P = 2.71 × 10-5 and 1.58 × 10-5, respectively) but this was associated with acquisition of carfilzomib resistance, whereas there were no significant differences in *PSMB8* or *PSMB9* expression levels during disease progression in the TJP1-up patients (Table S1: PSMB8 209040_s_at and PSMB9 204279_at probe sets).

The molecular association between increased *TJP1* expression and TAZ/TEAD activation and exactly how this contributes to proteasome inhibitor resistance and the LP-1/Cfz-like high-risk MM phenotype remain to be clarified. A number of tight junction-associated signaling pathways have been described [63]. One possibility is that upregulated *TJP1* expression activates TAZ similar to that previously reported for the PDZ domain-containing tight junction protein PARD3 which involves protein phosphatase 1 [64]. Another candidate effector for future study is Rnd3/RhoE, a multifunctional Rho family GTPase that acts both as a regulator of the cytoskeleton and translation initiation [65, 66]. Rnd3 participates in focal adhesion and tight junction formation together with TJP1 (Figure 5C) [67] as well as in tumor cell migration [68, 69]. We previously identified *RND3* as a novel Nrf2-upregulated target in LP-1/Cfz cells associated with the translational reprogramming leading to carfilzomib resistance [8, 9].

In any event, our data indicate the importance of additional patient stratification when considering use of *TJP1* as a biomarker of proteasome inhibitor sensitivity in MM. On the other hand, our findings with translation inhibitors in LP-1/Cfz cells support further preclinical testing and potential clinical evaluation of these agents in relapsed/refractory MM.

## MATERIALS AND METHODS

### Cell culture

Carfilzomib-resistant LP-1/Cfz cells have been described previously [8]. RPMI-8226 cells were obtained from the American Type Culture Collection (Manassas, VA). Doxorubicin-resistant RPMI-8226/Dox40 cells [31], melphalan-resistant RPMI-8226/LR5 cells [32] and mitoxantrone-resistant RPMI-8226/MR20 cells [33] were kindly provided by Dr. William Dalton (Moffitt Cancer Center, Tampa, FL). Cells were cultured in RPMI 1640 with GlutaMAX (Thermo Fisher Scientific) supplemented with 10% fetal bovine serum (Cambrex BioScience), 100 U/ml penicillin and 100 μg/ml streptomycin. Cultures were maintained at 37°C in a humidified atmosphere containing 5% CO2.

### Antibodies and reagents

The following antibodies were used: anti-ZO-1/TJP1 (ZO1-1A12) (Fisher Scientific, Cat. No. 33-9100); anti-TAZ (V386) (Cell Signaling Technology, Cat. No. 4883); anti-TEF-1(TEAD1) (Clone 31/TEF-1) (BD Biosciences, Cat. No. 610922); anti-Nrf2 (H-300) (Santa Cruz Biotechnology, Cat. No. sc-13032); anti-MCL1 (S-19) (Santa Cruz Biotechnology, Cat. No: sc-819); and anti-α-tubulin mouse mAb (DM1A) (EMD Millipore Corporation, Cat. No. CP06). Carfilzomib (Cat. No. A-1098) was obtained from Active Biochem; emetine (Cat. No. 32-469-3250MG) was purchased from Fisher Scientific; and homoharringtonine (omacetaxine mepesuccinate) (Cat. No. SML1091) was from Sigma-Aldrich.

### Confocal microscopy

Immunofluorescence confocal microscopy was performed essentially as described previously [7, 8]. In brief, cells were plated onto poly-L-lysine-coated microscope slides (Thermo Fisher Scientific, Cat. No. 50-279-88) in 10 cm cell culture dishes (5 × 10^5^ cells in 1 ml of medium per slide) [70]. After 3 hours, 10 ml of medium was added and the cells were cultured overnight. Attached cells were fixed in 3.7% formaldehyde for 5 minutes at room temperature and permeabilized with 0.5% Triton X-100 in phosphate-buffered saline (PBS) for 15 minutes at room temperature. Following permeabilization, the cells were rinsed with PBS and blocked in PBS containing 10% goat serum and 0.01% Triton X-100 for 40 minutes at room temperature. The cells were then incubated with anti-TJP1 and anti-TAZ antibodies diluted 1:500 in PBS containing 1% goat serum and 0.01% Triton X-100 for 1 hour at room temperature. The cells were rinsed three times with PBS and then incubated with Alexa Fluor 488-conjugated goat anti-mouse (Thermo Fisher Scientific, Cat. No. A-11001) and Alexa Fluor 568-conjugated goat anti-rabbit (Thermo Fisher Scientific, Cat. No. A-11036) secondary antibodies diluted 1:500 in PBS containing 1 μM TOTO-3 (Thermo Fisher Scientific, Cat. No. T3604), 1% goat serum and 0.01% Triton X-100 for 1 hour at room temperature. The cells were rinsed with PBS and mounted with Fluoromount G (Electron Microscopy Sciences). Imaging analysis was performed on an LSM 710 laser scanning confocal microscope equipped with Zen software (Carl Zeiss Microscopy).

### siRNA transfections

RNA interference was performed as described previously [8] using validated Silencer® Select siRNAs for WWTR1/TAZ (s24789) [37], TEAD1 (s13961) [38], YAP1 (s20366) [38], TJP1 (s14155, s14156) [54, 55] or a negative control siRNA (Cat. No. 4390843) (Thermo Fisher Scientific). Briefly, 2 × 10^6^ cells per ml were seeded into 24 well plates in 100 μl aliquots. Each well received a mixture of siRNAs (750 ng) and HiPerFect reagent (6 μl) in 100 μl serum-free culture medium preincubated for 15 minutes. After 5 hours, the cells were diluted to 6 × 10^5^ per ml in complete medium. Cells (3 × 10^5^ per ml) were seeded into 96 well plates and treated with a range of carfilzomib concentrations.

### Cytotoxicity assay

Cells were treated with carfilzomib and agents at the indicated concentrations and cell growth was measured using the alamarBlue cell viability and proliferation reagent (Thermo Fisher Scientific) as described previously [6-8], except that assays were performed on fibronectin-coated plates (Fisher Scientific, Cat. No. 08-774-60) [71].

### Gene expression analysis

Gene expression profiling data for LP-1/Cfz and parental LP-1 MM cells is available at GEO: GSE78069. Datasets for MM patients are available at GEO: GSE2658 and GEO: GSE31161.

### Gene set enrichment analysis (GSEA)

GSEA [26] was performed using the MSigDB v5.2 collections. Statistical significance of the enrichment score was assessed by 1,000 gene set permutations.

## SUPPLEMENTARY MATERIALS FIGURE AND TABLE




