# Supplementary material for: Increased expression of the tight junction protein TJP1/ZO-1 is associated with upregulation of TAZ-TEAD activity and an adult tissue stem cell signature in carfilzomib-resistant multiple myeloma cells and high-risk multiple myeloma patients
Source: Oncoscience. 2017 Aug 1;4(7-8):79–94. doi: 10.18632/oncoscience.356 (PMC5616201; doi:10.18632/oncoscience.356)

SUPPLEMENTARY MATERIALS

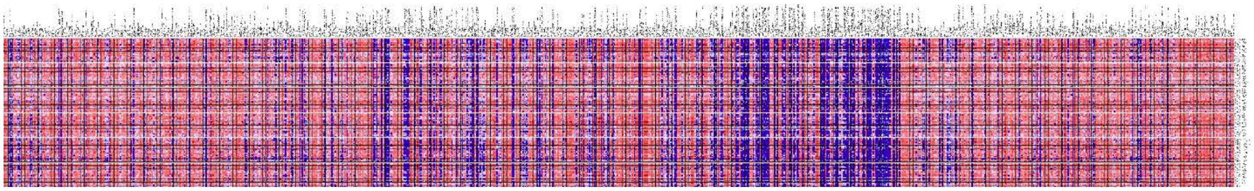

Figure S1: The top 20 neighbors of WWTR1/TAZ, TEAD1, TJP1, ITGB5, CRIM1 and YAP1 across 947 cell lines in the the Cancer Cell Line Encyclopedia (see the following page for an expanded view). WWTR1/TAZ, TEAD1, TJP1, ITGB5, CRIM1 and YAP1 are indicated with an asterisk on the right side of the figure.

Table S1: Please see separate Supplementary Table file.

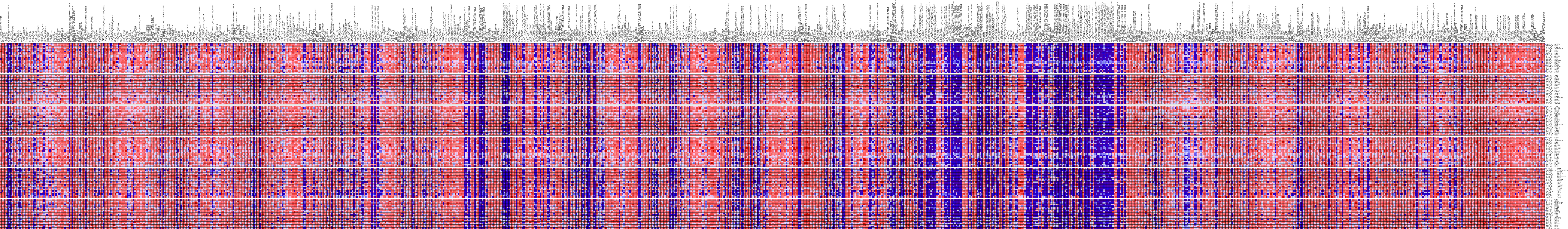

Supplement: Supplementary file 1 [file oncoscience-04-0079-s001.pdf]
